# A Pilot Study of Enterade (VS001), an Oral Amino Acid Formulation, in Malnourished Bangladeshi Children with Environmental Enteric Dysfunction

**DOI:** 10.4269/ajtmh.24-0402

**Published:** 2025-02-11

**Authors:** Masud Alam, Tahsin Ferdous, Rifat Ara, Abdulla Siddique, Mamun Kabir, Rashidul Haque, Jeffrey R. Donowitz

**Affiliations:** ^1^ Infectious Diseases Division, International Centre for Diarrhoeal Disease Research, Bangladesh, Dhaka, Bangladesh;; ^2^ Vaccine Testing Center, University of Vermont, Burlington, Vermont;; ^3^ University College London, London, United Kingdom;; ^4^ Division of Pediatric Infectious Diseases, Children’s Hospital of Richmond at Virginia Commonwealth University, Richmond, Virginia;; ^5^ Division of Pediatric Infectious Diseases, University of Virginia, Charlottesville, Virginia

## Abstract

Environmental enteric dysfunction (EED) is a subacute syndrome characterized by increased intestinal inflammation and permeability that affects children in low-income countries. It is associated with growth and neurodevelopmental deficits, and there is currently no known treatment for EED. VS001 (AmiLyfe Bioscience, LLC, Norwood, MA) is a medical food (beverage) containing free amino acids that has been shown to decrease enteric inflammation and improve gut permeability in murine models. We conducted a double-blind, placebo-controlled pilot study to assess the acceptability and tolerability of VS001 in Bangladeshi children aged 1–2 years (*n* = 10 per arm). We also examined the effects on EED biomarkers (lactulose–mannitol (LM) ratio, fecal lactoferrin, alpha-antitrypsin, myeloperoxidase, and neopterin). Participants received 8 oz. of VS001 or an identical vehicle without amino acids daily for 14 days. Tolerability and acceptability were measured using parental surveys and daily in-home adverse event monitoring. Subjects took an average of 118 minutes to complete the dose each day. Caregivers found the product convenient and easy to administer and either agreed or strongly agreed that they would give this product to their child again. None reported that the intervention negatively affected their child’s appetite. There were three mild adverse events deemed possibly related to the intervention, with two occurring in the active arm and one in the control arm. Children in the active arm exhibited a nonsignificant decrease in LM ratios (a marker of intestinal permeability) after treatment compared with the control arm (0.19–0.08 versus 0.19–0.17; *P* = 0.16). VS001 was acceptable to parents and reasonably well tolerated. Given the decrease in permeability observed in the active arm, a larger trial is warranted.

## INTRODUCTION

Environmental enteric dysfunction (EED) is a complex syndrome associated with living in unsanitary conditions in low-income countries. The syndrome involves the triangular interplay of enteric infection, enteric inflammation, and intestinal dysbiosis.^1^ It is ubiquitous, with almost all children in certain impoverished areas showing some evidence of chronic enteric inflammation, which is the hallmark of EED.^2^^,^^3^ Environmental enteric dysfunction has been documented in children as young as 8 weeks of age.^4^ The association between EED and subsequent growth delays is well described.^2^^,^^5^^–^^8^ Although pathogen carriage and associated inflammation likely play a role in EED pathogenesis, EED is a distinct entity from diarrheal disease.^7^^,^^9^^–^^11^

Although the gold standard for diagnosing EED is endoscopy and histologic examination of the small intestine, noninvasive biomarkers for the condition have been established. Dual sugar tests, including the lactulose–mannitol (LM) ratio, measure small intestinal permeability and are widely used as biomarkers of EED.^12^ Fecal biomarkers of absorption and inflammation have also been used to assess EED. Among the numerous tests available, fecal lactoferrin, fecal myeloperoxidase, fecal neopterin, and fecal alpha-1 antitrypsin are among the most studied.^7^^,^^13^^,^^14^

Currently, there is no known treatment for EED. Although the implementation of adequate sanitation throughout the world remains a top priority, the treatment of EED is also essential. The lack of effective therapy for EED represents an immense knowledge gap in efforts to improve childhood health worldwide. Novel therapies aimed at preventing colonization by pathogenic enteric bacteria, reducing enteric inflammation, repairing intestinal barrier integrity, and restoring a healthy intestinal microbiota are needed, particularly for infants and toddlers, because the effects of EED are most severe in this population and carry the highest morbidity.

VS001 (AmiLyfe Bioscience, LLC, Norwood, MA), marketed under the trade name Enterade, is a medical food designed to improve intestinal integrity. It was originally developed for the treatment of radiation- and chemotherapy-induced enteropathy in cancer patients, a condition that has significant clinical and pathological overlap with EED.^15^ VS001 contains a proprietary formulation of five amino acids (valine, aspartic acid, serine, threonine, and tyrosine) selected for their ability to improve intestinal absorption and integrity.^16^ Preclinical work suggests that VS001 decreases intestinal apoptosis, intestinal permeability, bacterial translocation, and intestinal interleukin-1 beta-mediated inflammation while increasing epithelial proliferation and brush border protein expression in mice exposed to radiation.^16^^–^^18^ An analysis of messenger RNA expression indicates that these results are partially due to the restoration of claudin-1, -3, and -7 expression by epithelial cells.^18^

A recent retrospective analysis demonstrated that VS001 reduced the frequency of bowel movements in patients with neuroendocrine tumors, for whom diarrhea can be debilitating.^19^ Furthermore, a randomized placebo-controlled analysis of VS001 in autologous hematopoietic stem cell transplant patients undergoing high-dose chemotherapy showed a significant reduction in diarrhea in patients in the active trial arm after 14 days (16% versus 86%; *P* <0.03).^20^ VS001 has also been tested in pediatric populations; however, the results of these efforts have not been published.

Given the growing body of literature demonstrating the positive effects of VS001 on intestinal permeability and symptoms of enteropathy, we conducted a pilot interventional trial to determine if VS001 was acceptable and tolerable in an urban Bangladeshi population with a high burden of EED. Biomarkers of EED were measured as part of an exploratory analysis.

## MATERIALS AND METHODS

We conducted a double-blind, randomized, placebo-controlled trial to determine the safety, acceptability, and tolerability of VS001 compared with a placebo in Bangladeshi children aged 1–2 years with increased LM ratios. Ten children were enrolled in each arm. This sample size was selected to provide data to power a subsequent trial with the LM ratio as the primary outcome while minimizing the pilot sample size, given the lack of data on this product’s tolerability in malnourished children. The placebo was an identical product without the proprietary formulation of amino acids. Both products were packaged in individual-serving 8 oz. bottles, which were identical in appearance and flavor (orange-flavored). The bottles were labeled only with “Group A” or “Group B.” Both the study product and placebo were obtained from AmiLyfe Bioscience, LLC (Norwood, MA). Both were stored in a temperature-controlled cabinet at 22°C according to the manufacturer’s recommendation (18°C to 25°C).

A community census of the Dhaka North City Corporation, Bangladesh, specifically in wards 2, 3, and 5 within the Mirpur neighborhood, was conducted through door-to-door canvassing. Children between 1 and 2 years of age whose parents expressed interest in the study underwent anthropometric screening. Those with a length-for-age Z score (LAZ) between –1 and –2 SD underwent LM testing using previously published methods.^21^ Briefly, children were fasted for a minimum of 2 hours before consuming a solution containing 250 mg/ml of lactulose (Osmolax, Square Pharmaceuticals Ltd, Dhaka, Bangladesh) and 50 mg/ml of mannitol (Sigma, St. Louis, MO) at a dose of 2 mL/kg body weight, with a maximum dose of 20 mL. Urine was then collected by using a pediatric urine collection bag for a minimum of 2 hours or until at least 2 mL of urine was produced. Children were allowed to return to their regular diet 30 minutes after completing the dual sugar solution. One to two drops of thimerosal (Sigma, St. Louis, MO) were added to the samples before freezing them at –80°C until the sugar concentration was assessed via liquid chromatography with tandem mass spectrometry. Twenty children with an LM ratio >0.09 were enrolled and randomized to either the active arm or the control arm, provided they had no current diarrheal symptoms and did not have a sibling previously enrolled. Lactulose–mannitol ratios were batched and run weekly. The LM ratio and LAZ criteria were specified to increase the likelihood that the enrolled children had symptomatic EED. Randomization was conducted using the R package “randomizeR” version 3.0.1 (R Foundation, Vienna, Austria). The randomization key was kept at Virginia Commonwealth University (Richmond, VA), and the field team was provided with sealed opaque envelopes labeled with the randomization sequence and containing a card with the randomization sequence and study arm assignment. Subjects, parents, and the Bangladeshi study team were blinded.

Subjects were visited daily by the study team and observed as parents administered the 8 oz. product daily for 14 days through directly observed therapy. Only the first dose was given in the study clinic to monitor for any immediate adverse effects. Each day, the child had a maximum of 8 hours to complete the dose. The study team conducted a tolerability questionnaire with the primary caregiver daily. This questionnaire was adapted from the pediatric-patient reported outcome-common terminology criteria for adverse events Caregiver Questionnaire (https://healthcaredelivery.cancer.gov/pro-ctcae/). At the end of the 14 days, an acceptability questionnaire was completed by the primary caregiver.

The LM test was repeated on day 15. Stool samples were collected on day zero (before starting the intervention) and again on day 15. The samples were stored at 4°C in our study clinic and then transported to the parasitology laboratory at the International Centre for Diarrheal Disease Research, Bangladesh within 4 hours of collection. There, they were stored at –80°C until they were removed for batch testing of fecal biomarkers using ELISA. The biomarkers tested included lactoferrin (Eagle Biosciences Inc., Nashua, NH), myeloperoxidase (Eagle Biosciences Inc., Nashua, NH), alpha-1 antitrypsin (Eagle Biosciences Inc., Nashua, NH), and neopterin (Biomatik, Wilmington, DE).

Responses to the tolerability and acceptability questionnaires were summarized using descriptive statistics. Because the study product and the control product were identical except for the presence of amino acids, acceptability data were analyzed for the entire cohort without accounting for the study arm. Using a repeated measures analysis of variance (ANOVA) test and a per-protocol analysis (i.e., the one subject who missed 4 days of the intervention in the control arm was removed from our analysis), the EED biomarkers were compared. SPSS version 27 (IBM, Inc Armonk, NY) was used for the analysis.

## RESULTS

**Figure 1.**
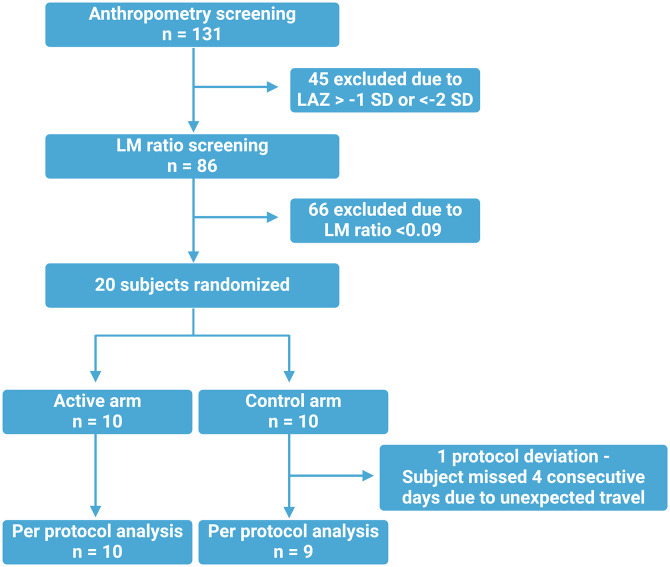
Consort diagram. Overall, 131 children were screened, with 10 enrolled per arm (total *N* = 20). There was a single protocol deviation, and our primary analysis was per protocol with that subject excluded.

A total of 131 children were screened for anthropometry, and 86 of those had a baseline LM test. Twenty children who met the enrollment criteria were randomly assigned (Figure 1). Screening and enrollment occurred in July and August of 2022. Key enrollment characteristics were similar in both arms, with the exception of the number of people living per room, which was higher in the control arm compared with the active arm (4.0 versus 2.6, respectively; *P* ≤0.001; Table 1). There was a single protocol deviation in which a subject in the control arm did not receive the study product for 4 consecutive days because of an unexpected death in the family, which resulted in that child traveling outside of Dhaka during that time.

**Table 1:** Baseline characteristics

Variable	Active Arm (*n* = 10)	Control Arm (*n* = 10)	*P*-Value
Income (Bangladeshi taka)*	16,280 (±6,617.31)	14,300 (6,777.87)	0.52
Number of people living per room*	2.6 (±0.81)	4.0 (±0.77)	<0.001
Any maternal education^†^	7	9	0.58
Female^†^	4	3	1.00
Any food insecurity^†^	8	8	1.00
Toilet shared with other households^†^	5	7	0.65
Open sewer outside home^†^	7	4	0.37
Treated water^†^	1	3	0.58
Length-for-age Z score*	−1.97 (±0.57)	−2.19 (±0.61)	0.41

* Mean (SD).

^†^ Count.

### Tolerability.

Both the active and control products were well tolerated. The average time taken to complete the daily dose was 118 minutes (113 minutes in the active arm and 123 minutes in the control arm). The average actual volume consumed over the maximum 8-hour window was 235.99 mL in the active arm and 235.72 mL in the control arm, of the 237 mL (8 oz.) full dose.

There were ten adverse events (six in the active arm and four in the control arm) reported among eight children during the course of the study. Of these, seven were judged by a study physician to be unrelated to the intervention. The remaining three adverse events were considered possibly related to the intervention. These included seven loose stools within a 48-hour period in a child receiving the active product, five loose stools and one instance of vomiting over 24 hours in a child in the active arm, and two instances of vomiting over 24 hours in a child in the control arm. All adverse events were mild (i.e., they were easily tolerated and possibly required one dose of medication/treatment) and self-limited. No children experienced decreased appetite or abdominal pain during the 14-day intervention period.

### Acceptability.

**Figure 2.**
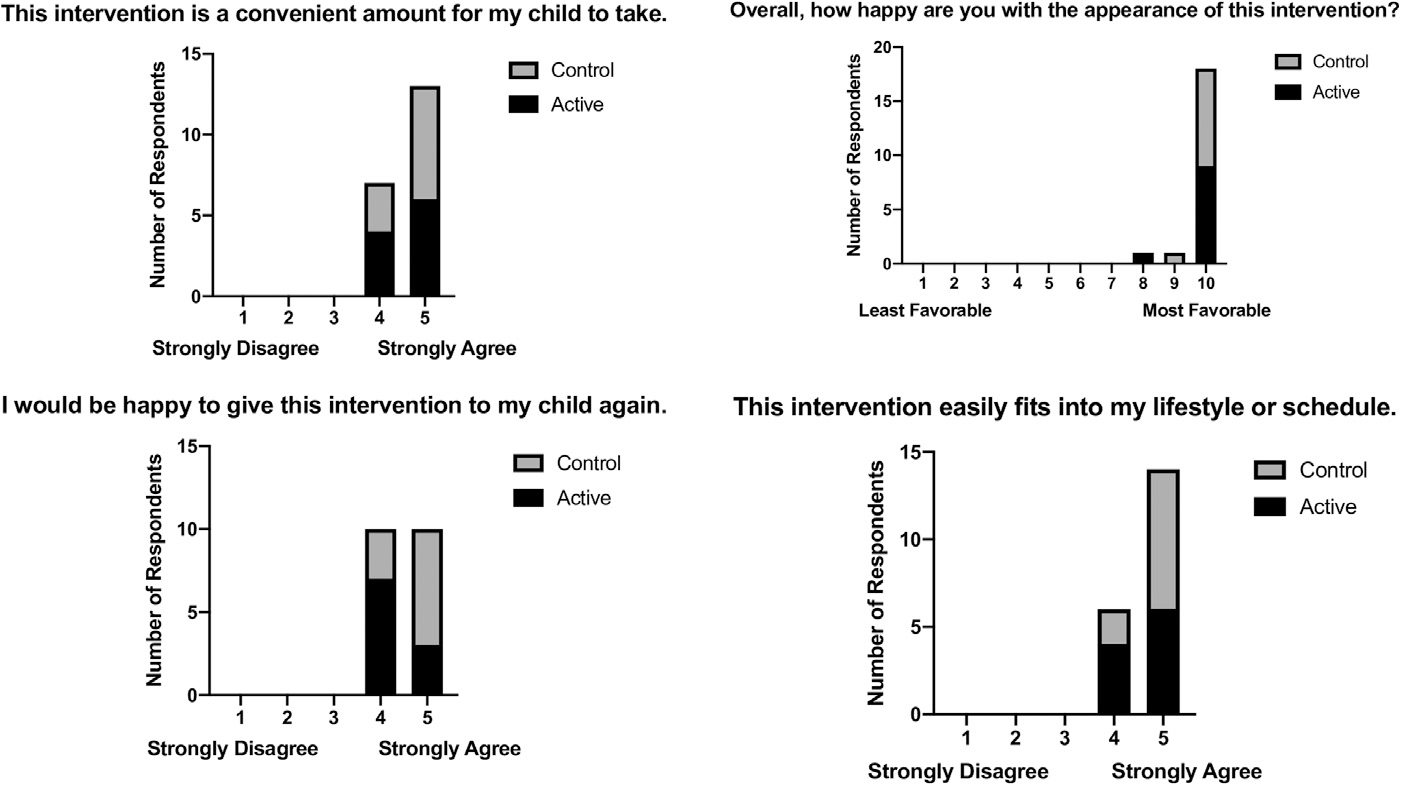
Responses to questions concerning the acceptability of the intervention were examined using a combination of the control and active arms. Overall, caregivers had a favorable impression of the intervention.

As the study product and the control product were identical except for the presence of amino acids, acceptability data were analyzed for the entire cohort without accounting for arm. Overall, the intervention was found by parents to be highly acceptable (Figure 2). Using a 1–5 scale (1 = strongly disagree, 2 = disagree, 3 = neither agree nor disagree, 4 = agree, 5 = strongly agree), parents rated the intervention as having convenient dosing (average 4.7), a convenient quantity (average 4.55), easy integration into lifestyle (average 5.0), and convenience of use (average 4.65). Parents also felt the product had a pleasant color (average 4.75), no bad smell (average 4.6), and appealing packaging (average 4.6) and was easy to administer (average 4.45). Parents were satisfied with the taste for their children (average 9.55, with 1 being least favorable and 10 being most favorable). All parents either agreed or strongly agreed that they would give this product to their child again. When asked about changes to their child’s appetite during the intervention period, nine of the twenty parents (four in the active arm, five in the control arm) felt that their child’s appetite increased, whereas the remainder felt it had no effect. All 20 children were still breastfeeding, although not exclusively. All 20 parents felt that breastfeeding was not altered by the intervention.

### Biomarkers of environmental enteric dysfunction.

**Figure 3.**
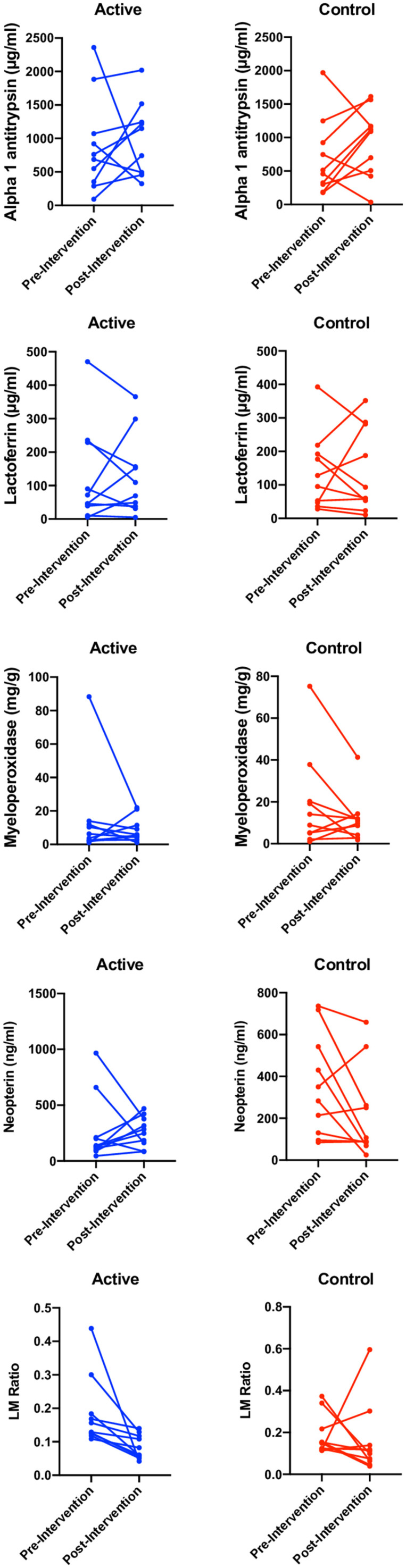
Biomarkers of intestinal inflammation were measured by using fecal ELISA pre- and post-intervention. These include lactoferrin, myeloperoxidase, and neopterin. The lactulose–mannitol (LM)  Figure 3 (*Continued*). ratio was measured via a dual sugar urinary excretion test using liquid chromatography with tandem mass spectrometry. No fecal markers were significantly different between time points. Changes in the LM ratio approached significance, with all children in the active arm normalizing their LM ratios. The average change in the active arm was –0.11, and in the control arm, it was –0.02 (repeated measures analysis of variance *P*-value = 0.16).

There was no significant difference in biomarkers between day 0 and day 15 when compared across arms (Table 2; Figure 3). However, there was a trend in the LM ratio, with an average change of –0.11 in the active arm and –0.02 in the control arm (repeated measures ANOVA *P*-value = 0.16).

**Table 2:** Environmental enteric dysfunction biomarkers

EED Biomarker	Active Arm (*n* = 10)		Control Arm (*n* = 9)		*P*-Value
	Pre-Intervention	Post-Intervention	Pre-Intervention	Post-Intervention	
LM ratio	0.19 (0.11)	0.08 (0.04)	0.19 (0.10)	0.17 (0.18)	0.16
Fecal myeloperoxidase (ng/mL)	14.20 (26.43)	8.79 (7.30)	19.43 (24.00)	11.72 (11.90)	0.17
Fecal alpha-1-antitrypsin (ng/mL)	896.82 (717.80)	963.70 (555.99)	622.14 (563.77)	869.67 (487.42)	0.37
Fecal neopterin (ng/mL)	265.50 (301.49)	263.60 (134.66)	366.67 (256.74)	238.78 (219.22)	0.32
Fecal lactoferrin (ng/mL)	124.39 (146.63)	127.59 (120.10)	146.06 (116.14)	149.86 (129.70)	0.90

LM = lactulose–mannitol. Presented as means (SD) and repeated measures analysis of variance *P*-values.

## DISCUSSION

This study demonstrated that VS001 had acceptable tolerability in young Bangladeshi children aged 1–2 years and was well accepted as a potential intervention by their caregivers. We showed that an 8 oz. daily intervention is feasible and does not interfere with dietary habits in this population. Despite our low sample size, an encouraging, although not statistically significant, trend was observed regarding improved intestinal permeability, as measured by the LM ratio in the active arm. However, no such trend was observed for the other EED biomarkers tested. This finding builds on the murine literature showing that a similar formulation of amino acids added to standard oral rehydration solution decreased electrophysiological conductance and paracellular permeability in the ileum compared with a saline control in a radiation enteropathy model.^18^

Currently, there are no accepted prevention strategies or therapies for EED. Several interventions to treat EED have been tested. Two large, rigorous cluster randomized trials of an intensive water, sanitation, and hygiene intervention were recently completed in Bangladesh and Zimbabwe. The Bangladeshi cohort experienced a significant decrease in diarrheal disease, intestinal permeability, and enteric inflammation, but this was not replicated in the Zimbabwe cohort. Linear growth was not affected in either cohort, although improvements in neurodevelopmental assessments were noted.^22^^–^^24^ A study of mesalamine in 1- to 5-year-old malnourished children in Kenya demonstrated no significant effect on markers of intestinal inflammation.^25^ In a multi-arm study of Zambian and Zimbabwean children aged 6–59 months, subcutaneous teduglutide showed a nonsignificant reduction in a composite score of EED biomarkers. Bovine colostrum, N-acetyl glucosamine, and budesonide showed no effect.^26^ A study of *Lactobacillus rhamnosus GG* in 3- to 5-year-old Malawian children demonstrated no effect.^27^ Promisingly, a recent trial of microbiota-directed complementary food in malnourished Bangladeshi children found a 0.01 SD increase in LAZ per week. Changes in the serum proteome did not suggest major effects on inflammation.^28^

Given the heterogeneity in EED, complementary therapies and prevention strategies are likely needed. Interventions aimed at decreasing enteric inflammation and improving intestinal permeability may enhance nutrition-based interventions by improving nutrient absorption. They may also contribute to improving gut barrier integrity in children with severe acute malnutrition, thereby decreasing commensal translocation, which is thought to be a driver of mortality in these children.^29^^,^^30^ Such adjunctive interventions may also complement water, sanitation, and hygiene preventive strategies. It is also possible that they play a primary role in EED prevention by mitigating the effects of colonization or infection with the pathogens thought to drive EED-associated inflammation.^31^^–^^33^ This work represents a first step toward the ability to trial VS001 in such circumstances, including providing the capacity to power future studies. Future trials will need to assess dosage, efficacy, and further evaluate both short- and long-term adverse effects that this trial was not powered to study.

This study had notable strengths. The intervention and placebo were administered through directly observed therapy, ensuring the reliability of our outcome data. In addition, our trial was double-blinded, placebo-controlled, and randomized, thereby limiting detection bias, performance bias, and selection bias. However, this study also had several important limitations that should be considered when interpreting our findings. First, the sample size was low, with only 10 patients per arm. Although we were able to assess trends in acceptability and tolerability, we did not have sufficient power to assess differences in biomarkers of inflammation or intestinal permeability. Second, our follow-up period was short, at 15 days, and we do not know if any effects of VS001 would be temporary or prolonged. Third, although VS001 demonstrated excellent acceptability in our study population, there are likely cultural influences on these measures that may differ in other regions of the world with pervasive EED. Although our acceptability and tolerability questionnaires were adapted from validated measures, they were not validated in our study population. Finally, although the documented adverse effects were few in number, not confirmed to be related to the intervention, self-limited, and minor, further trials will need to be adequately powered to more fully assess the adverse events associated with VS001.

## CONCLUSION

The acceptability and tolerability of VS001 in young Bangladeshi children aged 1–2 years was sufficient to warrant a larger and more prolonged trial to assess the impact of VS001 on EED. This work represents the first step in our ability to trial VS001 for EED in a variety of settings. Although there are challenges to scaling VS001 to address the burden of EED in young children worldwide (currently, Enterade retails for $4.50 per 8 oz. bottle), if effective, further work will need to be conducted to determine dosage, intervention timing, and duration, all of which may reduce the resources needed to use VS001 to treat EED.
